# Knockdown of Circ_0000144 Suppresses Cell Proliferation, Migration and Invasion in Gastric Cancer Via Sponging MiR-217

**DOI:** 10.4014/jmb.2102.02005

**Published:** 2021-05-05

**Authors:** Fengcun Ji, Chao Lang, Pengfei Gao, Huanle Sun

**Affiliations:** Department of General Surgery, Sunshine Union Hospital, High-Tech District, Weifang 261000, P.R. China

**Keywords:** Gastric cancer, circ_0000144, microRNA-217, proliferation, metastasis

## Abstract

Previous studies have uncovered the role of circ_0000144 in various tumors. Here, we investigated the function and mechanism of circ_0000144 in gastric cancer (GC) progression. The expression of circ_0000144 in GC tissues and cells was detected through quantitative real-time polymerase chain reaction (qRT-PCR) method. Gain- and loss-of-function experiments including colony formation, wound healing and transwell assays were performed to examine the role of circ_0000144 in GC cells. Furthermore, western blot was conducted to determine the expressions of epithelial mesenchymal transition (EMT)-related proteins. The interaction between circ_0000144 and miR-217 was analyzed by bioinformatic analysis and luciferase reporter assays. The circ_0000144 expression was obviously upregulated in GC tissues and cells. Silencing of circ_0000144 inhibited cell proliferation, migration and invasion of GC cells, but ectopic expression of circ_0000144 showed the opposite results. Moreover, circ_0000144 sponged miR-217, and rescue assays revealed that silencing miR-217 expression reversed the inhibitory effect of circ_0000144 knockdown on the progress of GC. Our findings reveal that circ_0000144 inhibition suppresses GC cell proliferation, migration and invasion via absorbing miR-217, providing a new biomarker and potential therapeutic target for treatment of GC.

## Introduction

Gastric cancer (GC) is one of the most common malignancies with high incidence and mortality [23, 27]. As a great threat to public health [18], the challenges of GC treatment are tumor recurrence and metastasis as well as non-specific treatment targets [3]. The occurrence and development of gastric cancer is a complex process involving multiple factors closely associated with the regulation of multiple genes. In recent years, surgery in combination with chemotherapy and radiotherapy, drug and molecular targeted therapies have been applied in the treatment of GC, but the 5-year survival rate of patients still remains less than 30% [10]. Many efforts have been made to improve the pathogenesis and treatment strategies of GC [5]. GC is generally diagnosed at late stage with a poor prognosis due to tumor metastasis [2], which poses a great challenge in improving the survival of gastric cancer patients [9]. Therefore, to further understand the biogenesis and pathogenesis of GC, it is highly urgent to improve diagnosis and develop new treatment strategies of GC.

The correlation between abnormal gene expressions of cancers and prognosis of patient has been applied to explain tumor progression. Circular RNAs (circRNAs) are a new type of ncRNAs and have covalently closed loop RNAs formed between 5′- and 3′-end [9, 22]. In recent years, circRNAs have been studied as transcriptional moderators through sponging microRNAs (miRNAs) in many types of tumors [14, 19].

CircRNAs, which are ubiquitously expressed in many tumor tissues [7, 12, 25], serve as a miRNA “cavernous body” to affect downstream target genes, and are involved in the development of various tumors [1, 11]. For instance, circRNA_001569 regulates the proliferation and invasion ability of colorectal cancer through targeting miR-145 [33]. Jian J *et al*. reported that dysregulated circRNA_100876 suppresses osteosarcoma cancer proliferation via targeting miR-136 [8]. In addition, circRNACER mediates breast cancer malignant progression by targeting the miR136/MMP-13 axis [24]. Mounting data have demonstrated that dysregulated circRNAs play critical roles in the progression of GC and may become a new target for GC diagnosis [21, 26, 29].

Hsa_circ_0000144 (also known as circSLAMF6), which is located at chromosome 1q23.2, is generated from the back-splicing of SLAMF6 first intron, and has been previously studied in many cancers [6, 30]. However, the biological function and the underlying mechanism of circ_0000144 in GC have not yet been investigated. Hence, we aimed to detect the expression of circ_0000144 in GC tissues and cell lines. Moreover, we explored the impact of circ_0000144 on GC proliferation and metastasis by applying gain- or loss-of-function experiments in vitro. Our findings may provide vital evidence for therapeutic approach and mechanisms underlying the pathogenesis of GC.

## Material and Methods

### Tissues Samples

The GC tissues and paired adjacent normal tissues samples were obtained from 45 patients who underwent surgery at Sunshine Union Hospital between January 2017 and December 2018. The research was carried out under the approval of the ethics committee of Sunshine Union Hospital, and informed consent was obtained from all patients. The tissues samples were immediately frozen at -80°C.

### Cell Culture and Cell Transfection

Normal human gastric epithelial cells (GES-1) and human GC cell lines (AGS, NCI-N87, SNU-5 and KATO III) were purchased from the Shanghai Center for Life Sciences Cell Center (China). The cell lines were cultured in DMEM (Gibco, USA) supplemented with 10% FBS (Gibco) at 37°C with 5% CO_2_ incubator.

The oligonucleotide small interfering RNA (siRNA) targeting circ_0000144 (si-circ_0000144), circ_0000144, miR-217 inhibitor (I) and matched negative vector (NC) (GenePharma, China) were individually transfected into AGS and NCI-N87 cells with a Lipofectamine 2000 Reagent Kit (Promega, USA) after reaching 80% confluence, following the manufacturer’s instructions.

### Quantitative Real-Time PCR (qRT-PCR)

Total RNA was extracted from tissues and cells using TRIzol reagent (Invitrogen, USA) and converted into cDNA using the M-MLV reverse transcriptase (Promega). The PCR reaction was performed on a Roche LC 96 qPCR System (Roche) with the following procedure: 95°C for 2 min, 40 cycles of 95°C for 10 s, and 60°C for 30 s. The specific primers were as follows: circ_0000144, forward: 5 -GAGTGTTGGCCTGTCCTCAA-3T, reverse: 5 -TTGTGCCCA GTTGCCTGTAT-3T, miR-217, forward: 5’-UACUGCAUCAGGAACUGAUUGGA-3′, reverse: 5’-CAAUCAGUUCCUGAUGCAGUAUU-3’; ANLN, forwards: 5’-CAAGATGTATCCAATGACT-3’, reverse: 5’-TGACTGAAGAATGAATGTT-3’. GAPDH or U6 were used as internal controls. The relative expression levels of genes were measured by the ^2−ΔΔCT^ method.

### Western Blot Analysis

The total protein was extracted by the RIPA lysis buffer and quantified using a BCA Protein Assay Kit (Takara). The proteins (30 μg) were loaded on 10% SDS-PAGE gels, separated by electrophoresis, and transferred into polyvinylidene fluoride (PVDF) membranes (Millipore, USA). The membranes were blocked with 5% non-fat milk and probed with primary antibodies at 4°C overnight. Next, the membranes were incubated in HRP-conjugated secondary antibodies for 1 h after washing in TBST 3 times. The protein bands were visualized by an enhanced chemiluminescence detection system (Bio-Rad Laboratories, USA). The primary antibodies were anti-E-cadherin (ab40772, abcam), anti-N-cadherin (ab18203, abcam), anti-Vimentin (ab92547, abcam) and GAPDH (ab8245, abcam).

### Colony Formation Assay

The transfected AGS and NCI-N87 cells (1 × 10^5^) were seeded into a 6-well plate. After culture for two weeks, the cells were fixed with methanol and subsequently stained with 0.1% crystal violet for 20 min. Finally, the colonies were counted under a light microscope.

### Scratch Wound Assay

The migration capability of GC cells was determined by wound scratch assay. AGS and NCI-N87 cells (1 × 10^5^) were seeded into a 6-well plate and held for 24 h. Scratches were created using a pipette tip, and the cells were incubated at 37°C for 48 h. Healing of the scratch was visualized at 0 h and 48 h under a light microscope. Each test was independently repeated at least 3 times.

### Transwell Assay

For invasion assay, the transfected cells (1 × 10^5^) were seeded in the upper chamber of transwell chambers (8 μm pores) pre-coated with 50 μl of Matrigel. Meanwhile, 20% FBS-DMEM was added to the lower chamber. The non-invading cells in the upper chamber were removed after cell culture for 48 h. Invading AGS and NCI-N87 cells were fixed with 4% paraformaldehyde for 15 min and stained with 0.5% crystal violet for 20 min (Sangon Biotech, China). The number of invasive cells was counted under a light microscope (Olympus, Japan) at least for 3 times.

### Luciferase Reporter Assay

Circular RNA Interactome (https://circinteractome.nia.nih.gov/) was used to predict binding sites between circular RNAs and miRNAs. The wild type or mutant report plasmid containing the 3’UTR of circ_0000144 was co-transfected with miR-217 inhibitor, NC, circ_0000144-WT or circ_0000144-MUT into cells using Lipofectamine 2000 Reagent (Promega). The luciferase activities were determined by a dual-luciferase assay system (Promega) after 48 h incubation.

### RNA Pull-Down Assay

Biotinylated RNA probes including Bio-miR-NC, Bio-miR-217-WT and Bio- miR-217-MUT were synthesized by KeyGen Biotech Company. These RNA probes were incubated with the lysates of AGS and NCI-N87 cells and extracted using streptavidin-coupled magnetic beads according to the instructions of Pierce Magnetic RNA Pull-Down Kit (USA). RNA-RNA complexes were then eluted using the salt solution, and purified using TRIzol (Pierce). The enrichment of ANLN in the RNA-RNA complexes was quantified using qPCR.

### Statistical Analysis

The monitoring data were analyzed by SPSS20.0 software (IBM, USA). Data were presented as mean ± SD. The significance of the differences was examined by Student’s *t*-test or one-way ANOVA, followed by Tukey’s post hoc test. *p* < 0.05 meant that the difference was significant.

## Results

### Circ_0000144 Was High-Expressed in GC Tissues and Cells

**Fig. 1:**
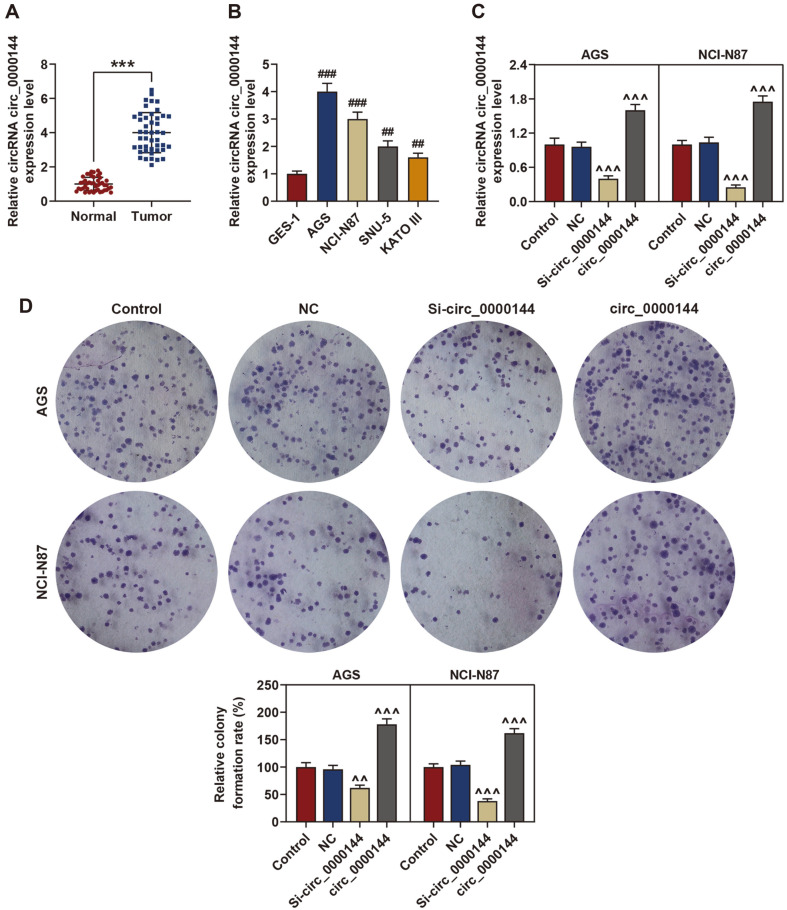
circ_0000144 was high-expressed in GC tissues and cells, and knockdown of circ_0000144 inhibits the proliferation of GC cells. (**A**) Circ_0000144 expression in GC tissues and matched adjacent normal tissues (*n* = 45). (**B**) Circ_0000144 expression level in GC cell lines. (**C**) The transfection efficiency was determined by qRT-PCR analysis. (**D**) Colony formation analysis was carried out to evaluate the effect of circ_0000144 on cell proliferation. ***p < 0.001 vs. Normal; ^##^*p* < 0.01, ^###^*p* < 0.001 vs. GES-1; ^^^*p* < 0.001 vs. NC. qRT-PCR: quantitative real-time PCR; GC: gastric cancer; NC, negative control.

To analyze the involvement of circ_0000144 in GC, we conducted qRT-PCR analysis to detect the circ_0000144 expression in GC tissues and paired adjacent normal tissues samples. The results indicated that compared with the matched normal group, the relative expression of circ_0000144 was obviously promoted in GC tissues (Fig. 1A). Additionally, circ_0000144 was also markedly higher expressed in four GC cell lines (AGS, NCI-N87, SNU-5 and KATO III) than in normal epithelial GES-1 cells (Fig. 1B). These results aroused our interest in further investigating the biological effects of circ_0000144 on GC.

### Knockdown of Circ_0000144 Inhibited Proliferation of GC Cells

To explore the biological function of circ_0000144 in GC, we transfected si-circ_0000144 and pcDNA3.1 circ_0000144 into AGS and NCI-N87 cells, and the transfection efficiency was verified by qRT-PCR assay. The data showed that circ_0000144 expression was obviously decreased after si-circ_0000144 transfection and was remarkably increased after circ_0000144 transfection, indicating that the transfection was successful (Fig. 1C). Colony formation analysis was applied to evaluate the effect of circ_0000144 on cell proliferation and the result demonstrated that the number of clones of AGS and NCI-N87 cells was reduced when circ_0000144 expression was decreased by contrast with the NC group, while the number of cell clones in the circ_0000144 group was obviously increased (Fig. 1D), suggesting that silenced circ_0000144 restrained the proliferation of GC cells.

### Silencing of Circ_0000144 Inhibited GC Cell Migration and Invasion In Vitro

**Fig. 2:**
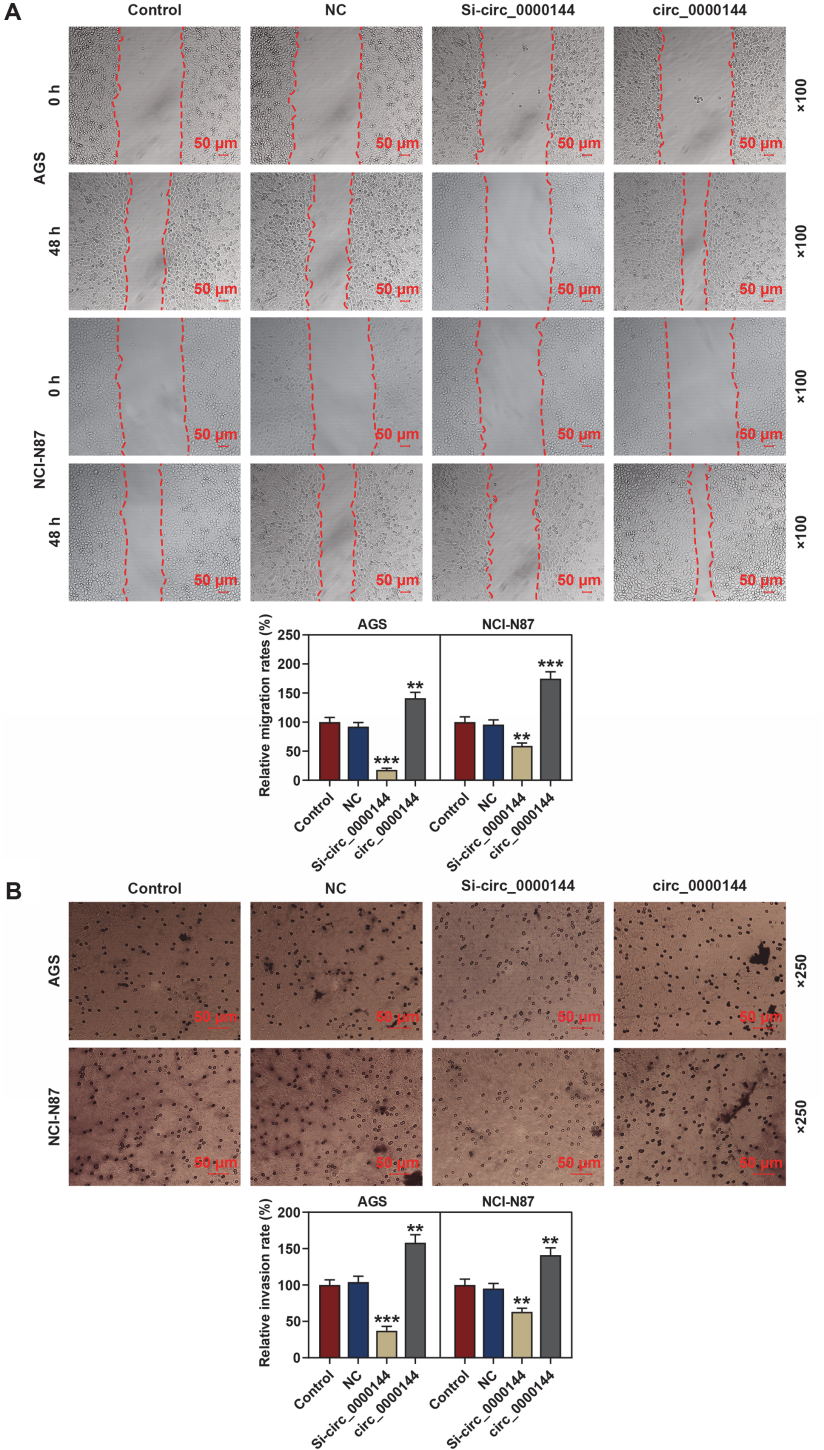
Silencing of circ_0000144 inhibited GC cell migration and invasion in vitro. (**A**) Wound healing assay was performed to investigate GC cell migration. (**B**) Transwell assay was conducted to explore the invasion of AGS and NCI-N87 cells. The experiment was independently conducted three times. **p < 0.01, ***p < 0.001 vs. NC. GC: gastric cancer; Sh, short hairpin RNA; NC, negative control.

To investigate the role of circ_0000144 in GC cell metastasis, we performed wound healing and transwell analysis. As shown in Fig. 2A, the result of wound healing assay showed that silencing circ_0000144 inhibited the migration of AGS and NCI-N87 cells, while circ_0000144 overexpression promoted the migration. The transwell assay result demonstrated that the number of invaded cells was significantly increased after circ_0000144 was overexpressed, however, the number of invaded cells was notably decreased in the circ_0000144-silencing group when compared with the NC group (Fig. 2B). These results suggested that transfection of circ_0000144 siRNA inhibited GC cell migration and invasion.

### Circ_0000144 Directly Sponged MiR-217

**Fig. 3:**
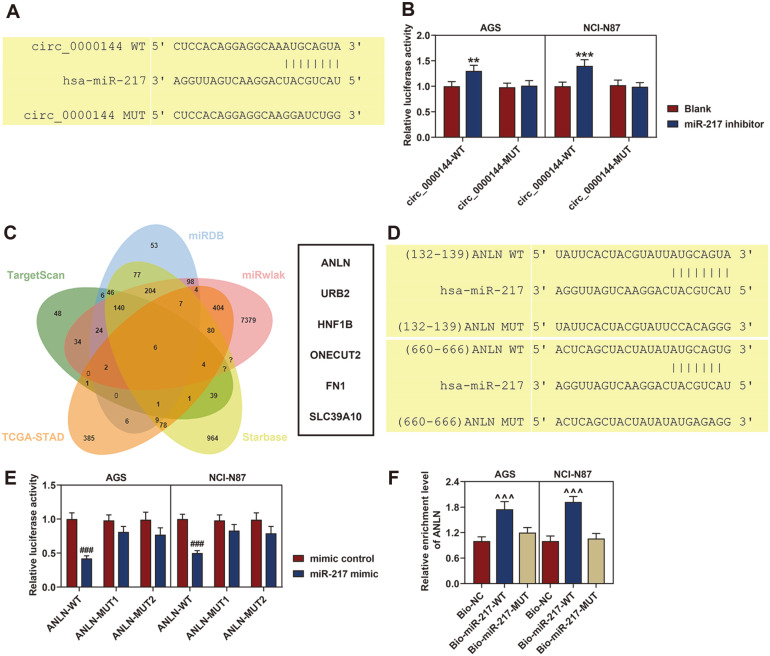
Circ_0000144 directly sponged miR-217. (**A**) The complementary binding site of miR-217 and circ_0000144. (**B**) Luciferase activity of circ_0000144-WT and circ_0000144-MUT after co-transfection with miR-217 inhibitor or NC in AGS and NCI-N87 cells. (**C**) The potential targets of miR-217 were predicted by TargetScan, miRDB, miRwalk, starBase and DEGS from TCGA-STAD database. (**D** and **E**) Luciferase report assay was performed to verify the interaction between ANLN and miR-217. (**F**) RNA pulldown confirmed the interaction between ANLN and miR-217. The experiment was independently conducted three times. **p < 0.01, ***p < 0.001 vs. Blank; ^###^*p* < 0.001 vs. mimic control; ^^^*p* < 0.001 vs. Bio-NC. WT: wild type; MUT: mutant type; GC: gastric cancer.

To explore the molecular mechanism of circ_0000144, we examined the potential target miRNAs of circ_0000144 using the CircRNA Interactome analysis. MiR-217 was identified as a potential target for circ_0000144, and the complementary binding site of miR-217 and circ_0000144 was presented in Fig. 3A. To verify the binding relation between circ_0000144 and miR-217, we performed the luciferase reporter assay using the circ_0000144-WT and circ_0000144-MUT plasmids. The results indicated that inhibition of miR-217 markedly enhanced the luciferase activity of circ_0000144-WT, but had no great effect on that of circ_0000144-MUT in AGS and NCI-N87 cells as compared with the blank group (Fig. 3B). These results revealed that circ_0000144 could act as a miRNA sponge for miR-217. Furthermore, the downstream targets of miR-217 were analyzed. In Fig. 3C, 6 genes were screened based on the prediction of miRDB, TargetScan, miRwalk, starBase and the DEGs in TCGA-STAD. The luciferase report further verified the interaction between miR-217 and ANLN (Figs. 3D and 3E). Moreover, the interaction was confirmed by RNA pull down assay, the ANLN enrichment in Bio-miR-217-WT group was higher than that in Bio-miR-217 Mut group, and miR-217 probes pulled down ANLN (*p* < 0.001, Fig. 3F). ANLN may function as a potential target of miR-217 in the promoting effects of circ_0000144 on GC development.

### Deletion of MiR-217 Expression Partly Reversed the Inhibitory Effect of Circ_0000144 Silencing in GC Cells

**Fig. 4:**
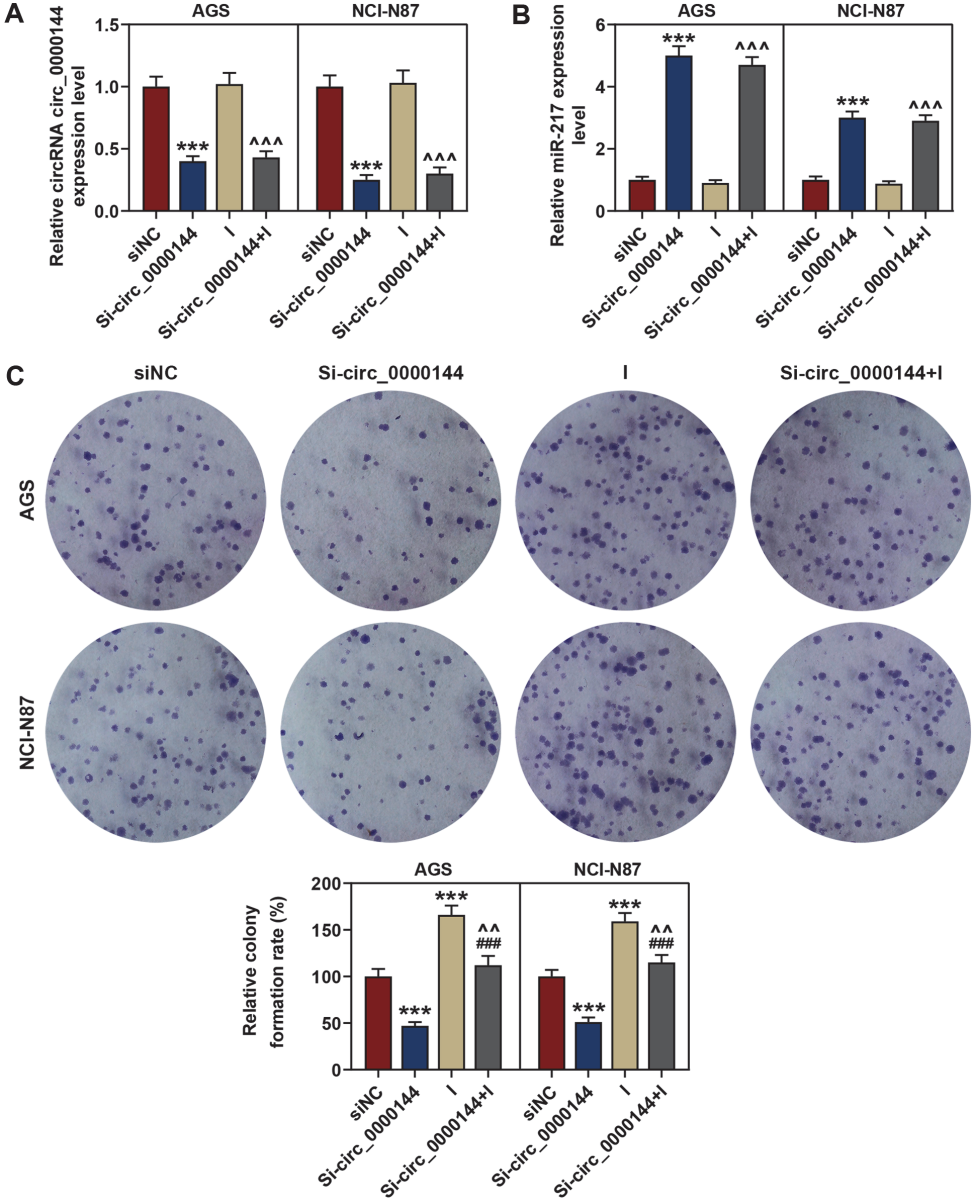
Inhibited miR-217 expression reversed the inhibitory effect of circ_0000144 silencing on GC cell proliferation. (**A, B**) The relative expressions of circ_0000144 and miR-217 were detected using qRT-PCR assay after transfection of si-circ_0000144, inhibitor or si-circ_0000144+ inhibitor in AGS and NCI-N87 cells. (**C**) The effects of circ_0000144 and miR-217 on cell proliferation were detected using colony formation analysis after transfection of sicirc_ 0000144, inhibitor or si-circ_0000144+ inhibitor in AGS and NCI-N87 cells. The experiment was independently conducted three times. ***p < 0.001 vs. siNC; ^###^*p* < 0.001 vs. Si-circ_0000144; ^^*p* < 0.01, ^^^*p* < 0.001 vs. I. GC: gastric cancer; NC, negative control; I: miR-217 inhibitor.

**Fig. 5:**
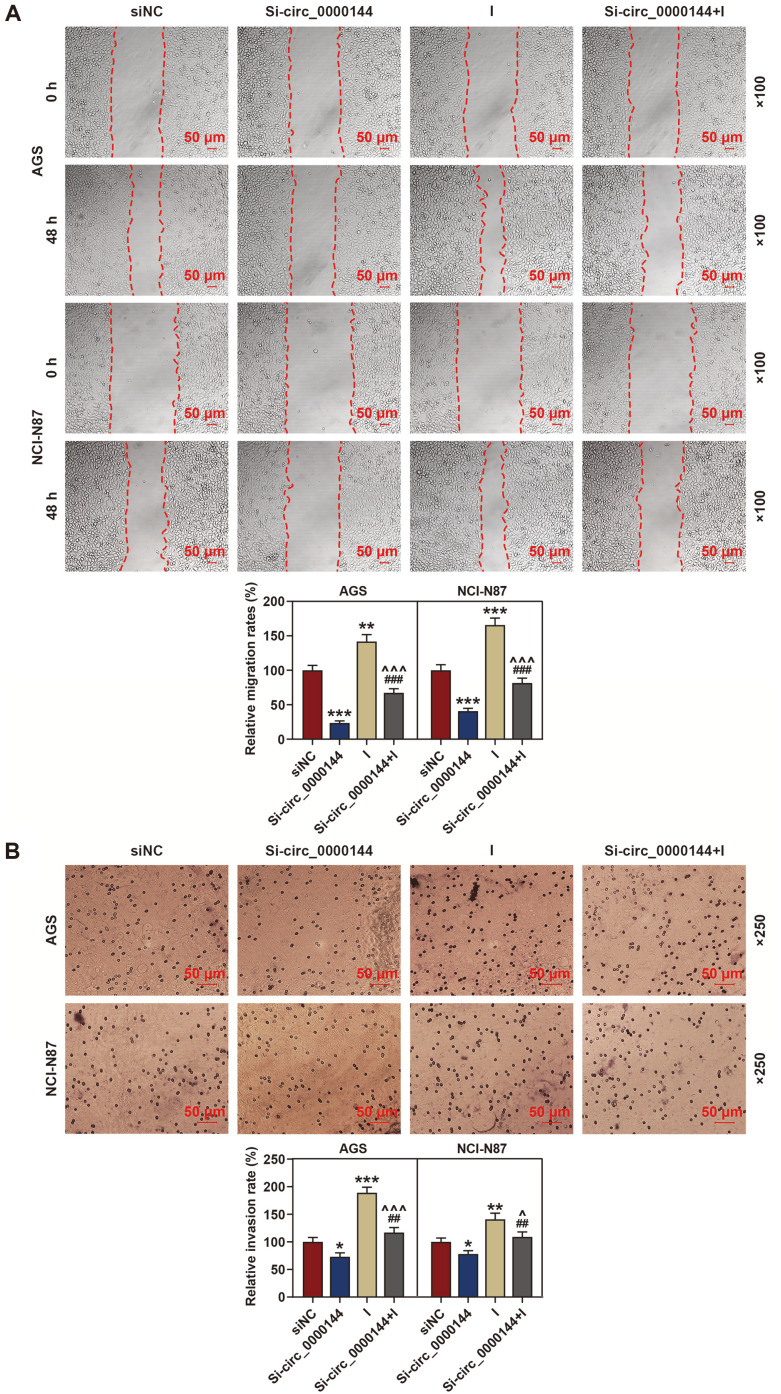
Inhibited miR-217 expression reversed the inhibitory effect of circ_0000144 silencing on migration and invasion in AGS and NCI-N87 cells. (**A**) Wound healing assay was performed to investigate GC cell migration. (**B**) Transwell assay was conducted to detect GC cell invasion. The experiment was independently conducted three times. *p < 0.05, **p < 0.01, ***p < 0.001 vs. siNC; ^##^*p* < 0.01, ^###^*p* < 0.001 vs. Si-circ_0000144; ^*p* < 0.05, ^^^*p* < 0.001 vs. I. GC: gastric cancer; NC, negative control; I: miR-217 inhibitor.

The regulatory relation of circ_0000144 and miR-217 in GC was investigated by rescue tests. To explore the effect of circ_0000144 on GC cells, the si-circ_0000144 and miR-217 inhibitor plasmids were co-transfected into AGS and NCI-N87 cells. The qRT-PCR analysis found that deletion of circ_0000144 induced the expression of miR-217, while miR-217 inhibitor had no effect on the expression of circ_0000144 when compared with the si-NC group (Figs. 4A and 4B). Next, colony formation assay results revealed that cell proliferation was notably reduced by silencing of circ_0000144, but was greatly restored by deletion of miR-217 (Fig. 4C). In addition, knockdown of circ_0000144 markedly inhibited cell migration and invasion, while silencing miR-217 significantly accelerated these processes when compared with the si-NC group (Figs. 5A and 5B).

**Fig. 6:**
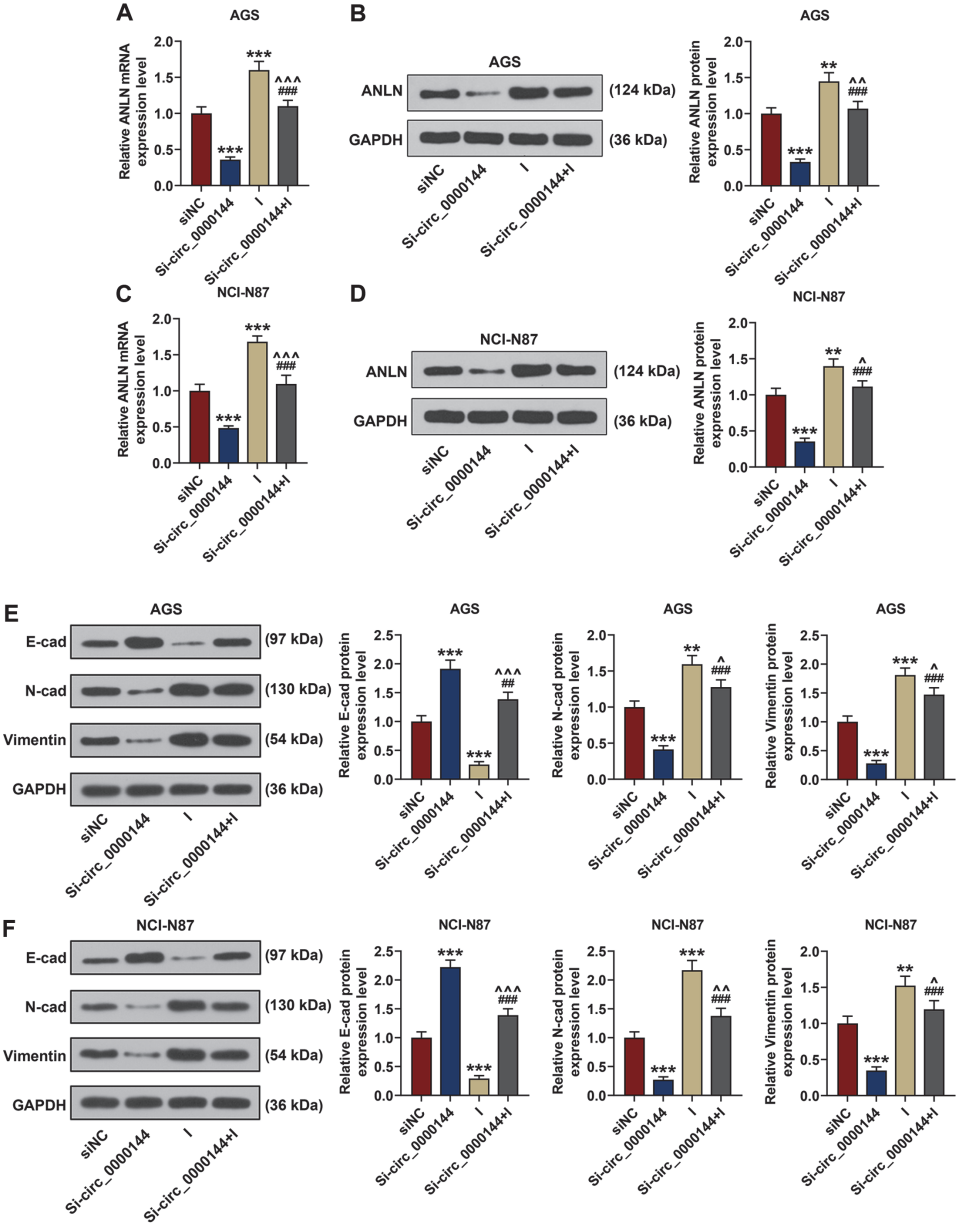
Inhibited miR-217 expression reversed the inhibitory effect of circ_0000144 silencing on epithelialmesenchymal transition (EMT)-relative proteins of GC cells. (**A-D**) The expressions of ANLN in AGS and NCI-N87 cells were measured by western blot and qRT-PCR analysis. (**E**) Expressions of E-cadherin, N-cadherin and Vimentin in AGS cells were measured by western blot analysis. (**F**) Expressions of E-cadherin, N-cadherin and Vimentin in NCI-N87 cells were measured by western blot analysis. GAPDH served as an internal reference. The experiment was independently conducted three times. **p < 0.01, ***p < 0.001 vs. siNC; ^##^*p* < 0.01, ^###^*p* < 0.001 vs. Si-circ_0000144; ^*p* < 0.05, ^^*p* < 0.01, ^^^*p* < 0.001 vs. I. GC: gastric cancer; negative control; I: miR-217 inhibitor.

Under the regulation of sicirc_0000144, the mRNA and protein expressions of ANLN were notably downregulated, while the transfection of miR-217 inhibitor could partially rescue the downregulated ANLN in circ_0000144-silent GC cells (Figs. 6A-6D). In addition, we also found that silencing circ_0000144 could downregulate the protein expressions of N-cadherin and Vimentin, while the effect was neutralized by inhibiting miR-217 expression (Figs. 6E and 6F). Moreover, E-cadherin protein expression was upregulated in AGS and NCI-N87 cells transfected with si-circ_0000144 when compared with si-NC group, but these effects were alleviated by miR-217 inhibitor, showing that the EMT was suppressed by circ_0000144 silencing and partly reversed by downregulated miR-217 in GC cells (Figs. 6E and 6F).

## Discussion

With the application of circRNA microarray technology, abnormally expressed circRNAs, which act as tumor suppressor or oncogenes, have been increasingly discovered in human tissues [4, 38, 39]. Evidence supports that circRNAs participate in various biological functions including proliferation, invasion, apoptosis, and metastasis of cancers. circRNA_104916 modulates EMT of colon cancer cells [20]. CircRNA LRP6 accelerates the development of osteosarcoma through regulating KLF2 and APC expressions [37]. Furthermore, Li *et al*. reported that circRNA_102958 facilitates the tumorigenesis of colorectal cancer through the miR-585/CDC25B axis [13]. circRNAs also contribute to the progression of of GC as indicated by previous reports. For example, circRNA_001569 facilitates GC cell proliferation via absorbing miR-145 [26]. Moreover, the study revealed that circPDSS1 could promote GC progression through modulating the miR-186-5p/NEK2 axis [21]. Wei *et al*. found that circ_0000144 was highly expressed in MGC-803 GC cells compared to GES-1 normal cells [30]. However, the regulating mechanism of circ_0000144 on GC cells is still unclear. In this study, we also proved that the expression of circ_0000144 was up regulated in GC tissues and cell lines, indicating that circ_0000144 might be a tumor-promoting factor in GC progression. However, whether the expression of circ_0000144 in these cell lines (AGS, NCI-N87, SNU-5 and KATO III) is higher than that of MGC-803 remains to be investigated.

Metastasis is induced by abnormal expressions of proto-oncogene and tumor suppressor genes, which will result in cancer cell proliferation, migration, and invasion[15]. Moreover, metastasis is also a major cause of cancer-related mortality, and EMT is a crucial driver of cancer malignancy and progression [16, 34]. The activation of EMT relies on the expressions of EMT-inducing transcription factors, such as Snail, Slug, E-cadherin, N-cadherin and Vimentin, which control epithelial and mesenchymal genes [17]. Functionally, we uncovered the function of circ_0000144 in GC, and revealed that knockdown of circ_0000144 inhibited cell proliferation and metastasis of GC cells.

To understand the molecular mechanism of circ_0000144 in metastasis, we determined the expressions of E-cadherin, N-cadherin and Vimentin, and found that knockdown of circ_0000144 up regulated the expression of the epithelial marker E-cadherin, but down regulated the mesenchymal markers N-cadherin and Vimentin. Therefore, we concluded that si-circ_0000144 inhibited cell ETM by regulation of E-cadherin, N-cadherin and Vimentin expression in GC cells. Thus, circ_0000144 was regarded as having an oncogenic role in progression of GC in the present study. Previous researchers have demonstrated that circRNAs can sponge miRNA to regulate cancer development [28, 32, 21]. Moreover, ircRNA_100269 has been found to suppress tumor cell growth by targeting miR-630 in GC [36]. Other findings also demonstrated that circ_0067934 promotes tumor growth and metastasis in hepatocellular carcinoma through regulation of the miR-1324/FZD5/Wnt/β-catenin axis [39]. In addition, Qu *et al*. reported that circRNACER mediates malignant progression of breast cancer through targeting the miR136/MMP13 axis [24]. Mechanistically, our bioinformatics analysis revealed that miR-217 bound with circ_0000144, which was further confirmed by luciferase reporter assay. The function of miR-217 had been demonstrated in many types of cancer. Previous studies proved that miR-217 acts as a potential tumor suppressor via regulating Wnt5a expression in osteosarcoma [31]. In addition, miR-217 affects cell growth and apoptosis through MAPK signaling pathway in colorectal cancer [35]. Further experiments revealed also that downregulation of miR-217 reversed the functions of circ_0000144 depletion on inhibiting cell proliferation, migration, invasion and EMT of GC. In addition, we also found that ANLN was targeted by miR-217, which inhibited the expression of ANLN. ANLN could function as a potential target of miR-217 in the promoting effects of circ_0000144 in GC development. However, our study also has limitations; for example, the specific regulatory mechanism of ANLN or downstream signaling pathway in GC requires further analysis.

To summarize, our current study verified that circ_0000144 serves in an oncogenic role to promote GC progression. Circ_0000144 is significantly up regulated in GC tissues and cells, and silencing circ_0000144 expression inhibits cell proliferation, migration and invasion through regulating miR-217. In addition, ANLN was the target gene of miR-217. Our findings provide a better understanding of the pathogenesis of GC and reveal that circ_0000144/miR-217 offers a potential therapeutic target for treatment of GC.

### Ethics Approval and Consent to Participate

The GC tissues and paired adjacent normal tissues samples were obtained from 45 patients who underwent surgery at Sunshine Union Hospital between January 2017 and December 2018. The research was carried out under the approval of the ethics committee of Sunshine Union Hospital, and informed consent was obtained from all patients.
